# Anatomical characteristics affecting the surgical approach of oblique lateral lumbar interbody fusion: an MR-based observational study

**DOI:** 10.1186/s13018-022-03322-y

**Published:** 2022-09-24

**Authors:** Meng-long Han, Wei-hong He, Zhong-yun He, Xue-liang Yan, Xiang-jun Fang

**Affiliations:** 1 Department of Radiology, Hengyang Medical School University of South China, The Second Affiliated Hospital, NO.30, Jiefang Road, Shigu District, Hengyang, Hunan 421001 China; 2 The Affiliated No.331 Hospital of ZhuZhou, Changsha Medical University, Zhuzhou, Hunan 412002 China; 3 Department of Orthopedics, Hengyang Medical School, University of South China, The Second Affiliated Hospital, Hengyang, Hunan 421001 China

**Keywords:** Oblique lumbar interbody fusion, Imaging anatomy, Preoperative evaluation, Magnetic resonance imaging

## Abstract

**Background:**

Oblique lateral lumbar interbody fusion (OLIF) is one of the most widely used lumbar interbody fusion procedures in clinical practice. The aim of this study was to minimize the incidence rate of surgical complications by measuring the anatomical parameters of structures surrounding the working channels of OLIF with 3D COSMIC sequence.

**Methods:**

The MRI examination included conventional MRI sequence and 3D COSMIC sequence. Surgical window, psoas thickness, the transverse diameter of the endplate, and nerve distance were measured to evaluate the anatomical characteristics surrounding the OLIF working channels.

**Results:**

The widths of the natural surgical window at the level of the L2–3, L3–4, and L4–5 intervertebral measured in this study were 16.25 ± 4.22, 15.46 ± 4.64 mm, and 11.71 ± 6.29 mm, respectively. The average thickness of the left psoas major muscle at the level of L2–3, L3–4, and L4–5 intervertebral space was 28.42 ± 5.08 mm, 30.76 ± 5.84 mm, and 31.16 ± 7.72 mm, respectively. The mean value of insertion angle (*β*) was 45.57° ± 6.19° in L2–3 intervertebral space, 49.90° ± 6.53° in L3–4 intervertebral space, and 43.34° ± 8.88° in L4–5 intervertebral space.

**Conclusions:**

The 3D COSMIC sequences can be used for imaging anatomical assessment before OLIF surgery. In preoperative planning, the 3D COSMIC sequence can be used to measure the relevant parameters mentioned above to optimize the planned surgical approach.

## Background

The incidence of lumbar degenerative disease (LDD) is increasing drastically with the increase in the number of aging patients. LDD is characterized by low back pain, radiating pain or numbness in the lower limbs and intermittent claudication, which can be further categorized as lumbar disk herniation, lumbar spinal stenosis, lumbar spondylolisthesis and kyphosis [1]. Conservative treatment such as immobilization and pain killers is usually used to relieve symptoms of LDD [2]. However, when symptoms keep worsening, lumbar interbody fusion (LIF) is usually employed to remove the pain surgically [3].

Oblique lateral lumbar interbody fusion (OLIF) is one of the most widely used lumbar interbody fusion procedures in clinical practice. OLIF can be traced back to the retroperitoneal approach that was first reported by Mayer in 1997 [4]. OLIF was introduced in China in 2014 and quickly accepted by clinicians. In OLIF, patients are held in a lateral recumbent position and the skin is cut with a small incision. After the dissection of the external oblique muscle, internal oblique muscle and transverse abdominis layer by layer, a working channel is built through the natural anatomical gap between the great retroperitoneal blood vessels and the anterior border of the psoas major muscle to reach the invertebrate region, where decompression, intervertebral body fusion and spinal fixation can be performed [5].

Although OLIF has many advantages, it is also associated with various complications during the operation [6, 7]. Improper maneuver during the operation may damage the abdominal arteries and veins, resulting in bleeding or hematoma, intraoperative violence and prolonged healing [8]. Furthermore, traction of the psoas major may cause damage to the lumbosacral nerve. As the operation needs to be performed through the retroperitoneal space, the peritoneum may also be easily damaged. Damage of the anatomic structural surrounding the working channel of OLIF can cause symptoms such as transient hip flexion weakness and sensory disturbance [9].

During the operation, the patient is placed in the lateral decubitus position and the median axillary line (that is, the coronal plane of the human body) is used as the baseline. The position of the surgical incision is established according to the placement angle measured by the preoperative MR image, so that the target intervertebral disk can be reached directly after the establishment of the surgical channel, avoiding the need for excessive stretch of the psoas muscle or compression of the abdominal aorta due to angle issues. To reduce or avoid complications caused by surgery, many studies have investigated the anatomy of structures surrounding the OLIF working channel. However, because of differences between autopsy research and actual surgical operations, the extrapolation of the results is difficult [10]. Most imaging studies are based on conventional magnetic resonance sequences and CT studies, which have disadvantages such as high ionizing radiation dose, low soft tissue resolution and poor lumbosacral nerve display [11].

The 3D COSMIC sequence is the GRE sequence based on T2WI multi-echo combining. The sequence uses a small-angle excitation pulse to collect 3–6 gradient echoes in the same phase encoding (same TR period), and all obtained data are collected [12]. Compared with conventional magnetic resonance sequences, the spatial resolution of this approach is improved, the susceptibility artifacts are reduced, and the nerve root as well as its anatomical relationship with adjacent tissues can be observed from multiple angles and directions.

In this study, we measured the anatomical parameters of structures surrounding the working channels of OLIF with 3D COSMIC sequence, with the aim to minimize the incidence rate of surgical complications of OLIF.

## Methods

### Demographic information of patients

MRI data from patients who received spine MRI examination due to limb numbness in a local hospital (The Second Affiliated Hospital, Hengyang Medical School, University of South China) from January 2020 to March 2022 were collected. The MRI examination included conventional MRI sequence and 3D COSMIC sequence. Among the 248 included patients, 124 were men and 124 were women, and the average age was 46.92 ± 12.56 years old. The research protocol was approved by the Ethics Committee of the local institute in accordance with its norms and standards (approval number: 201906).

Inclusion criteria included patients with typical symptoms of LDD, such as low back pain, leg pain, lower extremity weakness, numbness, and intermittent claudication; patients with intact MRI data; agreement to be included in the study; and a signed informed consent form for the study.

Patients who underwent abdominal surgery or lumbar spine surgery; patients with MRI showing abnormal development of lumbar vertebrae, such as butterfly vertebrae, hemivertebrae, lumbar sacralization, or sacral lumbar vertebrae; patients with MRI showing spinal deformity, such as scoliosis or kyphosis; patients with lumbar spondylolisthesis; and patients with lumbar infection, such as lumbar tuberculosis, and intervertebral disk infection were excluded from the study.

Magnetic resonance scanning was performed using a GE (GE Healthcare, Milwaukee, Wisconsin) Signa HDxT 3.0 T high-field-strength magnetic resonance scanner with 8-channel CTL spinal coil, and a post-processing workstation of GE AW4.6 version. The 3D coherent oscillatory state acquisition for the manipulation imaging contrast (COSMIC) sequence parameters are TR (5.7 ms), TE (2.8 ms), flip angle (40°), FOV (180 × 180 mm), matrix (288 × 288), slice thickness (3 mm), slice spacing (0 mm), number of slices (60), number of excitations (1), scanning range (L2-S1), scanning time (2 min and 34 s), and the scanning direction (from top to bottom).

### MR data evaluation and measurement

All magnetic resonance images were reviewed by two senior radiologists with specialty in musculoskeletal diagnostic imaging and one spinal surgeon. Data that did not meet the inclusion criteria were excluded. Measurements of anatomical parameters from L2 to L5 were performed by two radiologists. Scanning sequences included conventional sagittal T1WI, T2WI, STIR, axis T2WI and 3D COSMIC imaging sequences. The anatomical characteristics of left approach for OLIF surgery were measured and analyzed; most surgeons prefer to adopt the left approach, which is wider than its right counterpart.

### Anatomical parameters related to the OLIF surgery channel

**Fig. 1:**
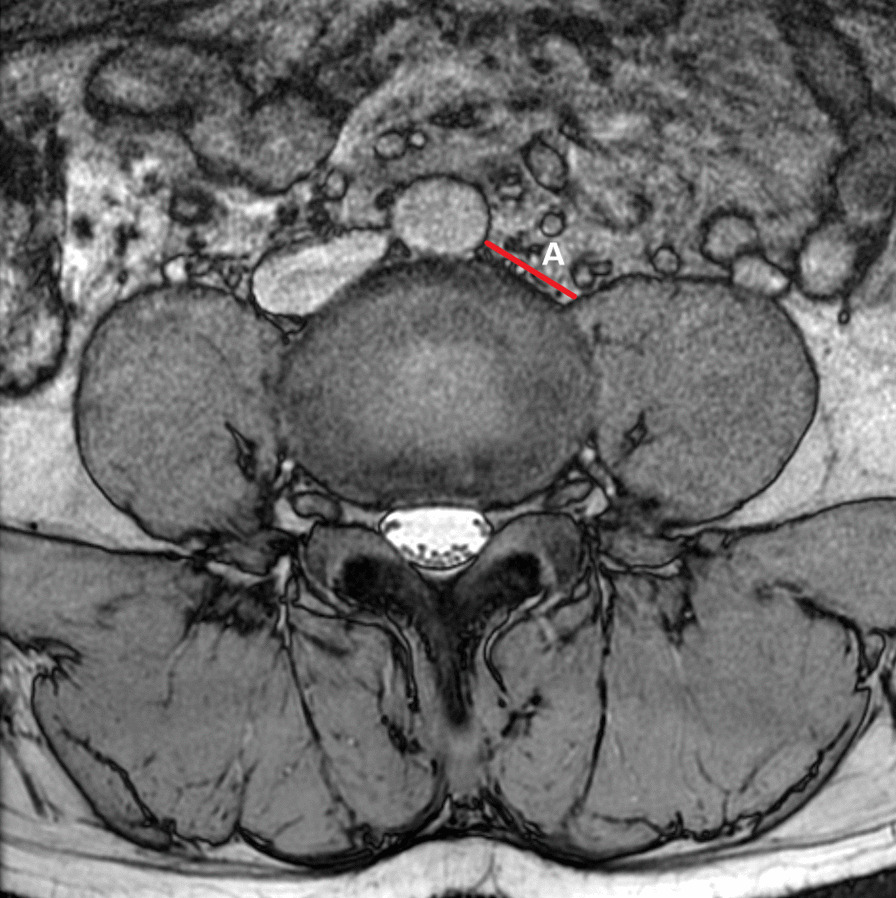
The width of the surgical window (A, red line): the shortest distance between the anterior border of the left psoas muscle and the abdominal aorta or left common iliac artery

**Fig. 2:**
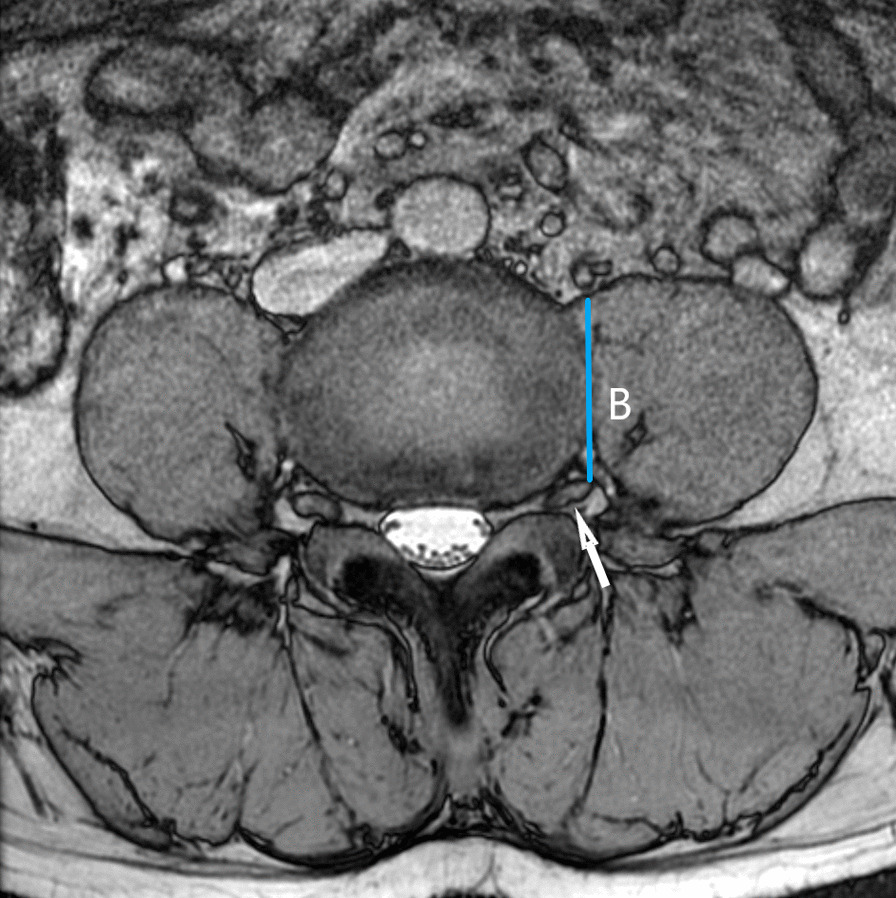
Psoas thickness: the distance (B, blue line) from the root of the left lumbar nerve (white arrow) to the anterior border of the left psoas muscle

**Fig. 3:**
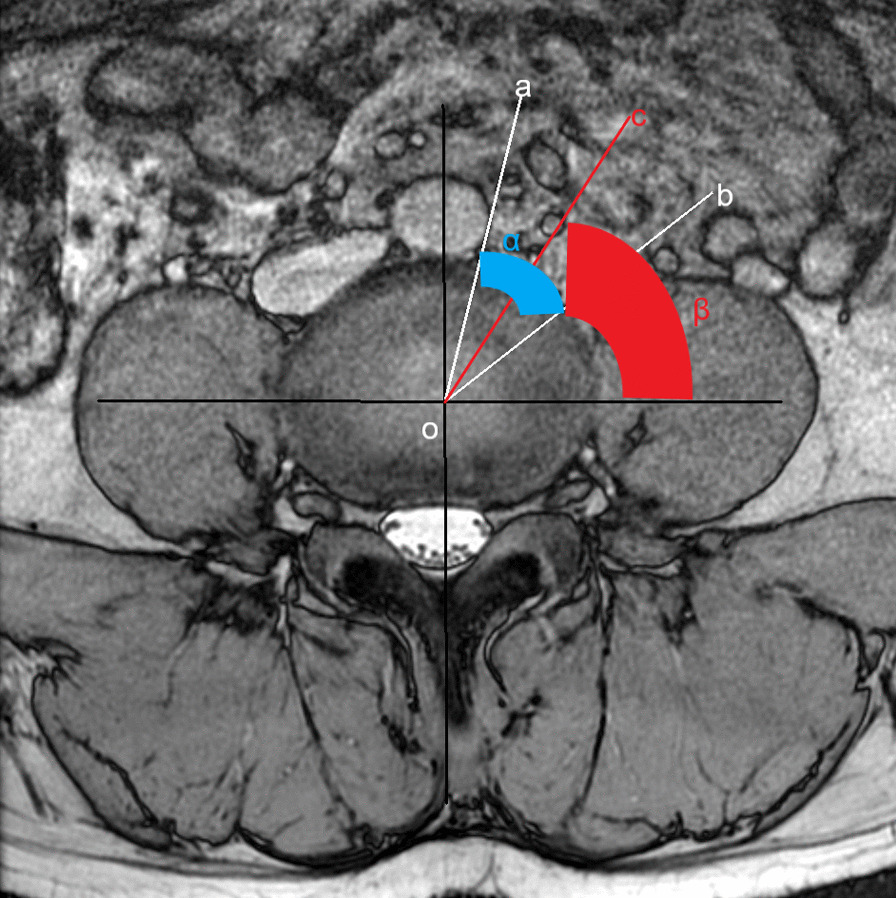
Insertion angle (*β*): Taking the intersection of the median sagittal plane and the median coronal plane of the intervertebral disk as point o, make the tangent line a between point o and the left side of the abdominal aorta (left common iliac artery) and tangent line b between point o and the front side of the left psoas muscle. The angle formed by the tangent line a and the tangent line b is ∠α. As the angle bisector c of ∠α, the angle formed by the c-line and the coronal diameter line of the median of the intervertebral disk is ∠*β*, which is the angle *β* when the OLIF surgical channel is placed

**Fig. 4:**
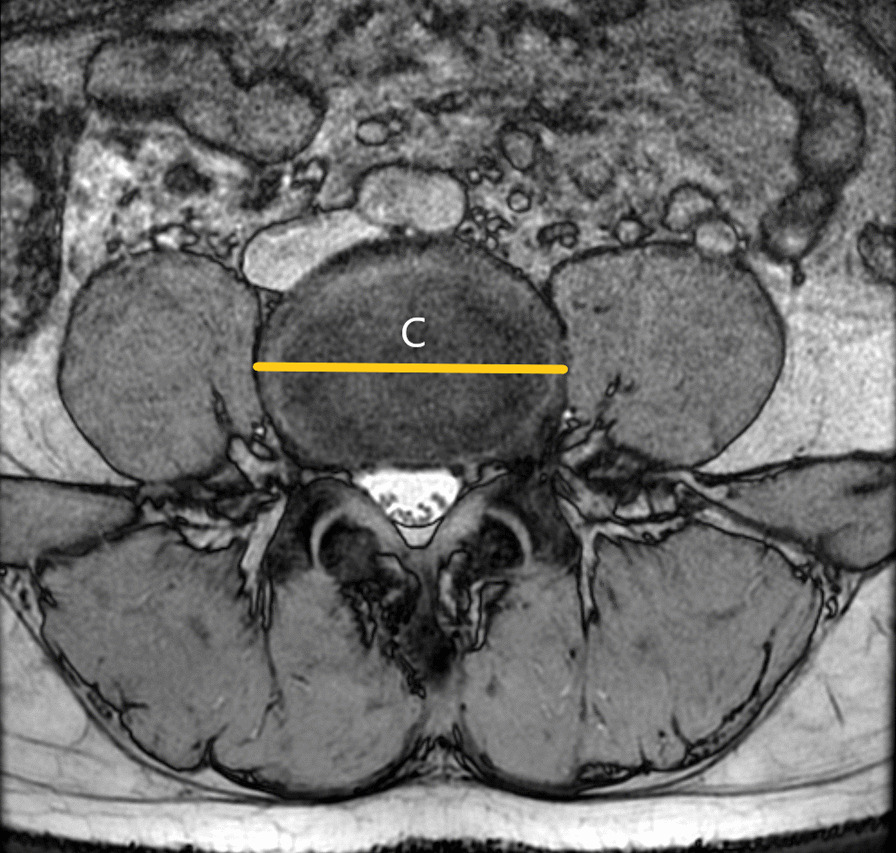
Endplate transverse diameter (C, yellow line): the maximum transverse diameter of the upper endplate of the vertebral body, which is defined as the length of the cage

**Fig. 5:**
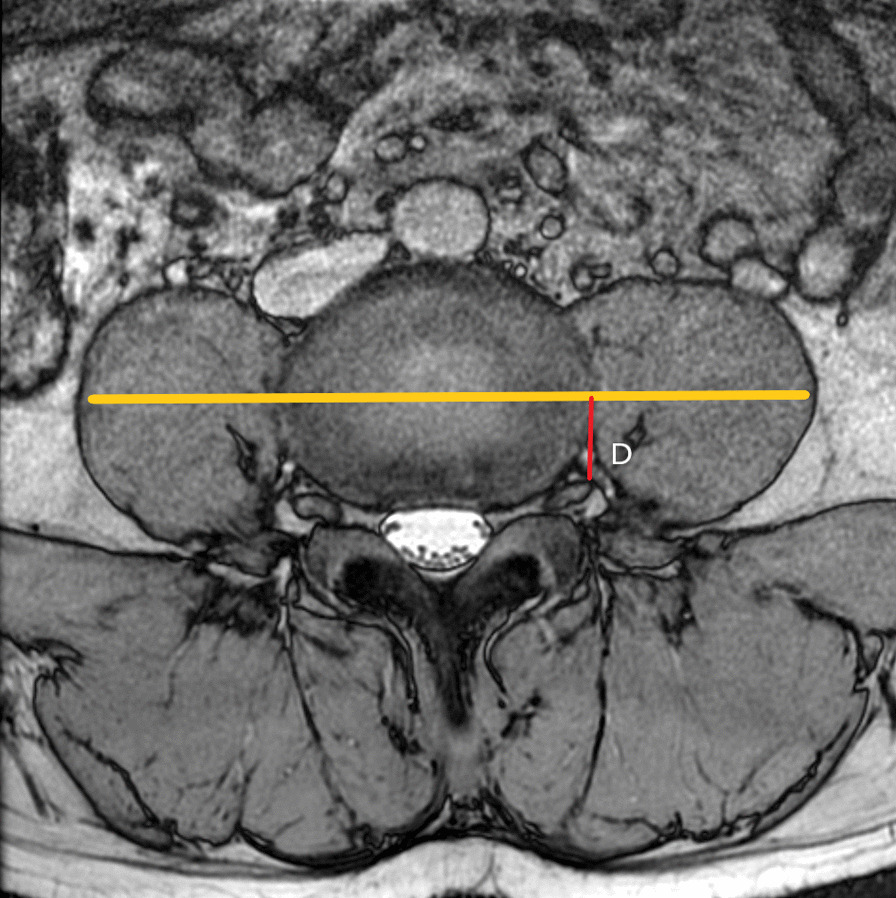
Distance (D, red line): sagittal distance from the anterior edge of the left lumbar nerve root to the coronal radial line passing through the median of the intervertebral disk

As shown in Fig. 1, the surgical window (A) was characterized by the shortest distance between the anterior border of the left psoas muscle and the abdominal aorta (or left common iliac artery) within each lumbar segmentation. Psoas thickness (B) was defined by the distance from the root of the left lumbar nerve to the anterior border of the left psoas muscle, as shown in Fig. 2. Insertion angle (*β*) was the formed by the c-line and the coronal diameter line of the median of the intervertebral disk, as shown in Fig. 3. The transverse diameter of the endplate (C) was defined as the maximum transverse diameter of the upper endplate of the vertebral body, which determines the length of the cage, as illustrated in Fig. 4. Nerve distance (D) was defined as the sagittal distance from the anterior edge of the left lumbar nerve root to the coronal radial line passing through the median of the intervertebral disk (Fig. 5).

### Statistical analysis

The intraclass correlation coefficient (ICC) test was used to evaluate the data consistency of radiologist 1 and radiologist 2 to measure the relevant anatomical parameters of the OLIF access channel (Table 1). One-way analysis of variance (one-way ANOVA) was used for the comparison of the anatomical parameters of the OLIF approach channel at different levels of the intervertebral space and the parameters of the channel placement angle. Normality test and Pearson correlation were used to analyze the correlation of anatomical characteristics with patient age, sex, and lumbar segment. A multiple regression analysis model was used to predict the factors affecting the insertion angle and surgical window. Chi-square test was employed for comparing the difference between the sexes.

**Table 1:** Consistency of measurement by two independent physicians

Measurement parameter	Sample size	Physician 1	Physician 2	ICC (95% confidence interval)
Surgical window	744	14.47 ± 5.50	14.59 ± 5.53	0.995 (0.994–0.996)
Psoas thickness	744	30.45 ± 6.49	30.52 ± 6.49	0.991 (0.990–0.992)
Insertion angle	744	46.60 ± 7.76	46.64 ± 7.80	0.989 (0.988–0.991)
Upper endplate transverse diameter	744	51.81 ± 4.47	51.79 ± 4.49	0.986 (0.983–0.988)
Distance D	744	13.29 ± 2.99	13.24 ± 3.03	0.971 (0.969–0.975)

## Results

### Comparison of surgical window among different vertebral segmentation

The width of the surgical window is one of the most important factors in evaluating whether OLIF surgery can be performed successfully. The widths of the natural surgical window at the L2–3, L3–4, and L4–5 intervertebral level measured in this study were 16.25 ± 4.22, 15.46 ± 4.64 mm, and 11.71 ± 6.29 mm, respectively. The width of the surgical window of the L3–4 intervertebral space in women was lower than that in men, as shown in Table 2. There were no significant differences between men and women in the remaining segments.

**Table 2:** Comparison of the surgical window among different vertebral segmentation

Intervertebral level	$\overline{x} \pm s$ (mm)		Men versus women		Surgical window
	Men	Women	*t*	*P*	
L2–3	15.91 ± 4.23	16.60 ± 4.21	− 1.297	0.196	16.25 ± 4.22
L3–4	14.74 ± 4.40	16.17 ± 4.79	− 2.446	**0.015**	15.46 ± 4.64
L4–5	11.66 ± 6.24	11.75 ± 6.35	− 0.105	0.917	11.71 ± 6.29

*P* value less than 0.05 was marked with bold and regarded as have statistical significant

Pearson correlation analysis between the width of surgical window and the intervertebral segment showed that r was − 0.337 and *P* < 0.001, which indicated that surgical window width decreased in lower vertebrae.

### Comparison of left psoas major muscle thickness among different vertebral segmentation

The average thickness of the left psoas major muscle at the L2–3, L3–4, and L4–5 intervertebral space was 28.42 ± 5.08 mm, 30.76 ± 5.84 mm, and 31.16 ± 7.72 mm, respectively. The thickness of the psoas major of men was greater than that of women, as listed in Table 3. Pearson correlation analysis was used to analyze the correlation between psoas thickness and intervertebral space, showing that r was 0.205 and *P* < 0.001.

**Table 3:** Comparison of left psoas major muscle thickness among different vertebral segmentation

Intervertebral level	$\overline{x} \pm s$ (mm)		Men versus women		Psoas major thickness
	Men	Women	*t *	*P*	
L2–3	31.30 ± 4.52	25.55 ± 3.34	10.792	** < 0.001**	28.42 ± 5.08
L3–4	34.45 ± 4.94	27.07 ± 4.09	12.805	** < 0.001**	30.76 ± 5.84
L4–5	36.90 ± 7.20	27.42 ± 4.75	12.240	** < 0.001**	31.16 ± 7.72

*P* value less than 0.05 was marked with bold and regarded as have statistical significant

### Comparison of insertion angle (*β*) among different vertebral segmentation

The mean value of insertion angle (*β*) was 45.57° ± 6.19° in L2–3 intervertebral space, 49.90° ± 6.53° in L3–4 intervertebral space, and 43.34° ± 8.88° in L4–5 intervertebral space, as listed in Table 4. The independent samples t test showed that in different lumbar spaces, the insertion angle in men was greater than that in women. Pearson correlation analysis between the placement angle and intervertebral level showed that r was − 0.143 and *P* < 0.001, which indicated that the placement angle decreased in lower intervertebral levels.

**Table 4:** Comparison of insertion angle (*β*) among different vertebral segmentation

Intervertebral level	$\overline{x} \pm s$ (°)		Male versus women		Insertion angle (*β*)
	Men	Women	*t *	*P*	
L2–3	48.22 ± 5.53	44.91 ± 6.40	4.361	** < 0.001**	45.57 ± 6.19
L3–4	52.73 ± 5.44	47.07 ± 6.31	7.572	** < 0.001**	49.90 ± 6.53
L4–5	46.95 ± 7.91	39.73 ± 8.33	6.995	** < 0.001**	43.34 ± 8.88

*P* value less than 0.05 was marked with bold and regarded as have statistical significant

### Comparison of the transverse diameter of upper endplate among different vertebral segmentation

The average transverse diameter of the upper endplate of the L3–L5 vertebral body was 49.38 ± 4.14 mm, 52.18 ± 4.05 mm, and 53.86 ± 4.03 mm, respectively. The independent samples *t* test showed that the transverse diameter of the superior endplate of the vertebral body in men was larger than that in women regardless of segmentation, as shown in Table 5. This suggests that the cage of men is larger than that of women during OLIF surgery. Pearson correlation analysis was used to analyze the correlation between the transverse diameter of the upper endplate of the vertebral body and the intervertebral space, showing that r was 0.410 and *P* < 0.001.

**Table 5:** Comparison of transverse diameter of upper endplate among different vertebral segmentation

Vertebra level	$\overline{x} \pm s$ (mm)		Male versus women		Transverse diameter of upper endplate
	Men	Women	*t *	*P*	
L3	51.28 ± 3.95	47.47 ± 3.40	8.128	** < 0.001**	49.38 ± 4.14
L4	53.81 ± 3.81	50.55 ± 3.62	6.917	** < 0.001**	52.18 ± 4.05
L5	55.43 ± 3.77	52.29 ± 3.66	6.660	** < 0.001**	53.86 ± 4.03

*P* value less than 0.05 was marked with bold and regarded as have statistical significant

### Comparison of distance D among different vertebral segmentation

The distances from the left nerve root to the midline of the intervertebral disk (referred as distance D) at the level of the L2–3, L3–4, and L4–5 intervertebral spaces were 14.94 ± 2.31 mm, 13.88 ± 2.61 mm, and 11.04 ± 2.55 mm, respectively, showing a decreasing trend from top to bottom, as shown in Table 6. At the L3–4 and L4–5 level, the distance D was greater in men than in women. Pearson correlation analysis was used to analyze the correlation between distance D and intervertebral space, showing that r was -0.571 and *P* < 0.001.

**Table 6:** Comparison of distance D among different vertebral segmentation

Intervertebral level	$\overline{x} \pm s$ (mm)		Men versus women		
	Men	Women	*t *	*p*	
L2–3	15.19 ± 2.18	14.68 ± 2.42	1.771	0.078	14.94 ± 2.31
L3–4	14.43 ± 2.43	13.34 ± 2.69	3.333	**0.001**	13.88 ± 2.61
L4–5	11.49 ± 2.33	10.59 ± 2.69	2.798	**0.006**	11.04 ± 2.55

*P* value less than 0.05 was marked with bold and regarded as have statistical significant

### The correlation of left psoas muscle thickness with surgical window and insertion angle.

**Fig. 6:**
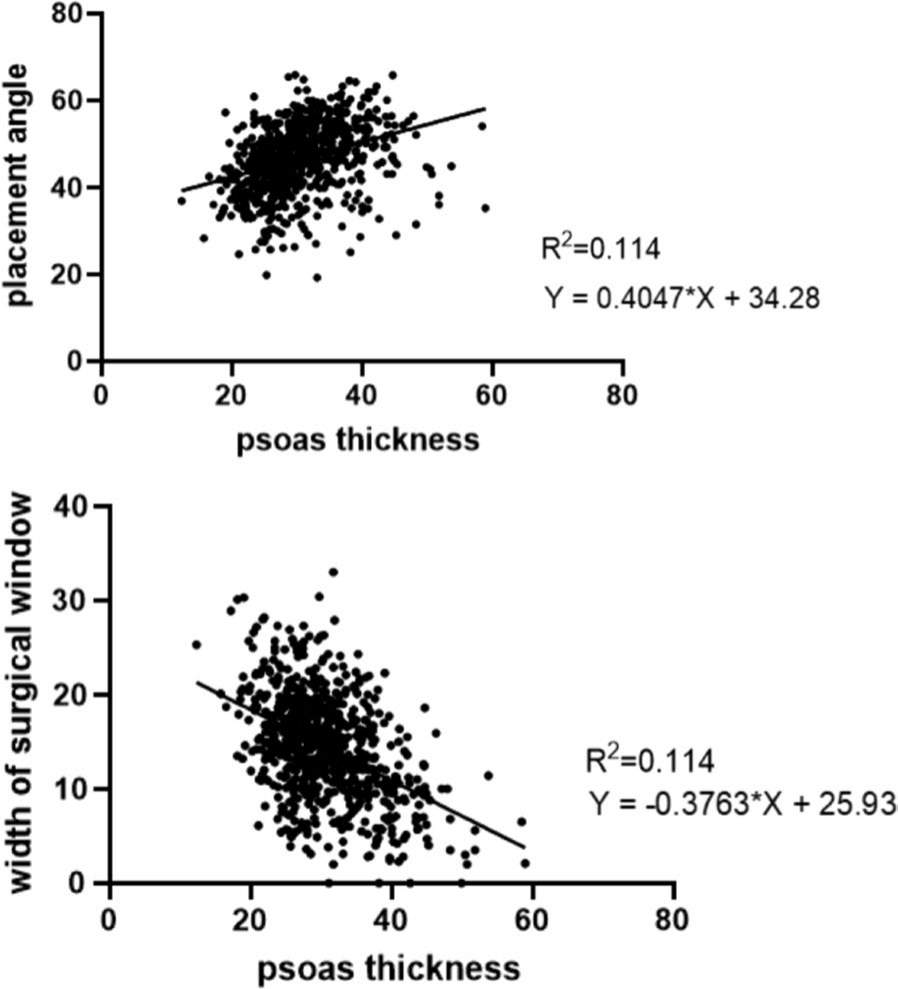
Pearson correlation of psoas thickness with placement angle and surgical window

The Pearson correlation between the width of surgical window and the thickness of the left psoas muscle was calculated, with R square as 0.114 and *P* < 0.001, which indicated that a thicker left psoas muscle could reduce the surgical window. Pearson correlation between the insertion angle and the thickness of the left psoas muscle showed that R square was 0.114 and *P* < 0.001, which indicated that thicker left psoas muscle could increase the insertion angle, as shown in Fig. 6. However, the R square with a low value indicated a low quality of correlation.

### Surgical window and related parameters

**Fig. 7:**
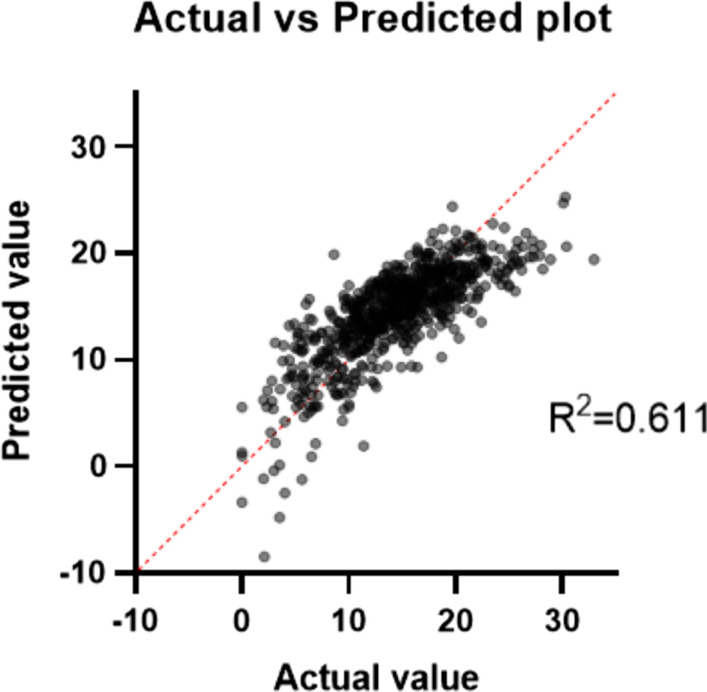
Prediction of surgical window using a multiple linear regression model. Surgical window = − 1.955 + 0.093*age − 1.112*sex − 0.5835*psoas major thickness + 0.2937* insertion angle + 0.2087* transverse diameter of vertebral superior endplate + 0.5256*distance D

Multiple linear regression analysis showed that the width of surgical window was correlated with age, sex, thickness of psoas major, distance D, and transverse diameter of vertebral superior endplate, as shown in Table 7 and Fig. 7.

**Table 7:** Parameters estimated in multiple correlation prediction of width of surgical window

Variable	Estimate	Standard error	95% CI (asymptotic)	|t|	*P* value
Intercept	− 1.955	2.225	− 6.324 to 2.414	0.8786	0.3799
Age	0.0933	0.01174	0.07026 to 0.1163	7.95	< 0.0001
Sex	− 1.112	0.3283	− 1.756 to − 0.4673	3.387	0.0007
Psoas thickness	− 0.5835	0.02585	− 0.6343 to − 0.5327	22.57	< 0.0001
Insertion angle	0.2937	0.01791	0.2586 to 0.3289	16.4	< 0.0001
Transverse diameter of vertebral superior endplate	0.2087	0.036	0.1381 to 0.2794	5.798	< 0.0001
Distance D	0.5256	0.04416	0.4389 to 0.6123	11.9	< 0.0001

### Insertion angle and related parameters

**Fig. 8:**
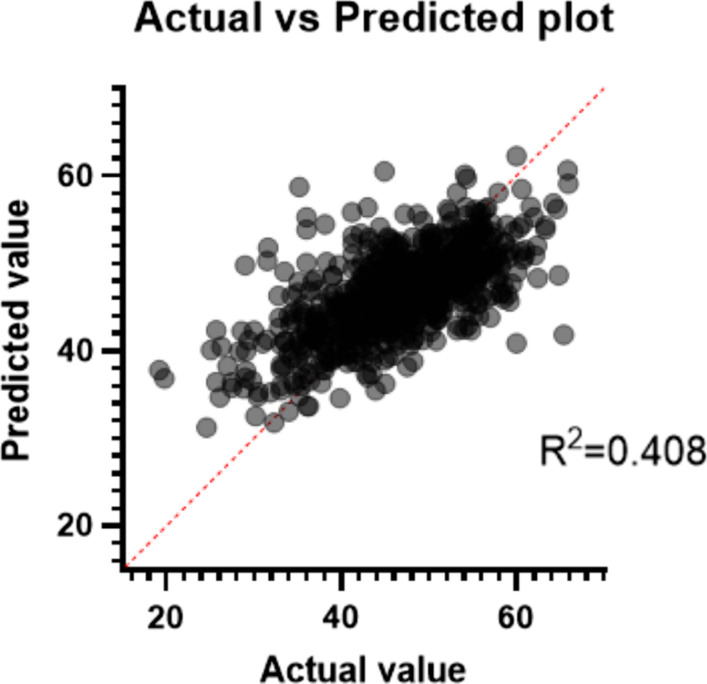
Prediction of insertion angle using multiple linear regression model. Insertion angle = 22.62–1.808*segmentation − 0.078*age + 0.875*width of surgical window + 0.775*psoas thickness − 0.378*distance D

Multiple linear regression analysis showed that the insertion angle was correlated with segmentation, age, psoas thickness, distance D and surgical window, psoas thickness, and distance D, as shown in Table 8 and Fig. 8.

**Table 8:** Parameters estimated in multiple correlation prediction of insertion angle

Variable	Estimate	Standard error	95% CI (asymptotic)	|t|	*P* value
Intercept	22.62	2.039	18.62 to 26.62	11.09	< 0.0001
Segmentation	− 1.808	0.34	− 2.475 to − 1.140	5.318	< 0.0001
Age	− 0.07746	0.01938	− 0.1155 to − 0.03942	3.998	< 0.0001
Width of surgical window	0.8752	0.05265	0.7719 to 0.9786	16.62	< 0.0001
Psoas thickness	0.7746	0.04072	0.6946 to 0.8545	19.02	< 0.0001
Distance D	− 0.3778	0.0946	− 0.5635 to − 0.1921	3.994	< 0.0001

## Discussion

MRI has been routinely used in the diagnosis, preoperative planning, and post-operative evaluation of lumbar spine diseases [13]. However, conventional MRI sequences show poor image quality of lumbosacral nerves and can only display lumbosacral nerves with limited range. The anatomical course of the nerve root cannot be observed in multiple directions and continuously. The 3D COSMIC sequence is a GRE sequence based on T2WI multi-echo combining. Compared with the conventional magnetic resonance sequence, the spatial resolution of this approach is improved; the artifacts are reduced, and the nerve root and its anatomical relationship with adjacent tissues can be observed from multiple angles [14]. In this study, a high-field strength 3.0 T MRI scanner was used to scan patients with low back pain by conventional magnetic resonance sequence and 3D COSMIC sequence to measure the anatomical parameters of the OLIF working channel.

The anatomical parameters related to the OLIF working channel and the insertion angle *β* measured by two radiologists were evaluated using the intra-group correlation coefficient ICC to ensure the consistency of the measurement results, as shown in Table 1. The tested ICC values of all parameters were all greater than 0.75. Therefore, the reproducibility of the data measured by the two investigators was satisfactory, and the relevant data could be used for further analysis. The data obtained by radiologist 1 were selected for the study.

OLIF is a surgical operation performed through the natural gap between the anterior edge of the psoas major muscle and the abdominal great vessels. The thickness of the psoas major is related to the difficulty of stretching the psoas major to extend the surgical field during OLIF surgery. A sufficient width of the natural operating window is a prerequisite for the operation [15]. In the present study, the width of operating window was defined by the shortest distance between the left psoas major muscle and the abdominal aorta. The width of operating window at the level of L2–3, L3–4, and L4–5 intervertebral space is 16.25 ± 4.22 mm, 15.46 ± 4.64 mm and 11.71 ± 6.29 mm, respectively. Davis et al. [16] conducted an autopsy study related to OLIF. The authors measured the access corridor diameters in 20 cadavers in the static state with the following findings: 18.60 mm at L2–3; 19.25 mm at L3–4; and 15.00 mm at L4–5. The results were close to the parameters measured in the present study. The Pearson correlation analysis between the width of surgical window and the intervertebral space showed that r was -0.337 and *P* < 0.001, which indicated that the surgical window width decreased in lower vertebrae. Women were slightly larger than men at the level of L3–4. For the remaining segments, the average distance between men and women was not statistically significant.

Safe and effective establishment of the surgical channel and adequate exposure of the target operating area are important steps in OLIF surgery. Establishing a surgical channel directly to the center of the target intervertebral space before surgery will make the surgical field more fully exposed, which is conducive to the operation under direct vision [17]. Therefore, it is very important to set up a proper insertion angle of the surgical channel to establish a surgical channel operated under direct vision. Mayer [4] measured the angle formed between the vertebral body and the operating table and made a 4 cm incision in front of the target intervertebral space in the same direction as the external oblique muscle fibers. Silvestre [18] made a 4 cm skin incision in the center of the target intervertebral space parallel to the ventrolateral area of the external oblique muscle fibers. This incision is perpendicular to the line from the anterior superior iliac spine to the umbilicus and is 1/3 away from the anterior superior iliac spine, similar to the McBurney incision.

In the present study, the L3–4 intervertebral level had the largest insertion angle of 49.90 ± 6.53°, while the insertion angle at the L2–3 intervertebral space level was 45.57 ± 6.19°. Notably, the insertion angles of different intervertebral space are different. The insertion angle in the channel in men was larger than that in women, and the results suggested that the incision position in men may be closer to the ventral side. The insertion angle and the thickness of the left psoas major showed a positive correlation. With the increase in the thickness of the psoas muscle, the insertion angle of the channel also increased.

The function of the cage is to accomplish fusion between the vertebral bodies to relieve the symptoms and maintain stability of the vertebrae [19]. The size of the cage used in OLIF surgery is vital for obtaining a long-term and stable curative effect. Improper positioning of the cage may lead to compression of the nerve root, subsidence of the cage or the collapse of the intervertebral space, which greatly impair the prognosis of surgery [20].

Cage subsidence is an important complication of OLIF surgery, which is closely related to the patient’s prognosis [21]. Reasonable selection of the size of the cage can reduce the probability of cage subsidence and achieve better bone graft fusion effect. In this study, the maximum transverse diameter of the endplate was used as the reference for selecting the cage size. In lumbar fusion surgery, the endplate of the surgical segment prevents the cage and bone graft from being embedded in the vertebral body, dispersing the stress, avoids the fusion of the cage, and promotes the fusion of the bone graft [22]. Zhang et al. [23] found that the cage with a length close to the outer region of the condyle ring of the endplate can achieve a larger area of biomechanical support and better prevent collapse of the vertebral body. In this study, the maximum transverse diameter of the upper endplate at L3, L4, and L5 vertebral bodies was measured to provide a reference for the size selection of the cage during OLIF surgery. The transverse diameter of the superior endplate of each vertebral body in men was larger than that in women. Pearson correlation analysis was used to analyze the correlation between the transverse diameter of the upper endplate of the vertebral body and the intervertebral space, showing that r was 0.41 and *P* < 0.001, which indicated that the transverse diameter of the upper endplate increased in lower levels. According to the results, it is recommended that when performing OLIF surgery, a cage with a length of 50–55 mm should be used for men and a cage with a length of 45–50 mm should be used for women. Chen et al. [24] defined the maximum transverse diameter of the intervertebral disk as the size of the cage. The measurements showed that the most commonly used cage lengths in clinical practice are 50 mm and 55 mm, which are similar to the results of this study.

In clinical practice, the nerve root is easily injured during the implantation of the cage, which may be caused by improper placement of the cage, incorrect size selection, and postoperative cage displacement and compression of the nerve root. In this study, the distance from the left nerve root to the median coronal line of the intervertebral disk was measured to provide a reference for the assessment of the risk of nerve root injury during cage placement. In this study, patients were scanned by 3D COSMIC neuroimaging sequence, and the distance from the left nerve root to the median coronal line of the intervertebral disk (distance D) was measured as the parameter characterizing the risk of nerve root injury during cage placement. According to our measurements, the distance D decreased as the number of intervertebral increased. Pearson correlation analysis was used to analyze the correlation between distance D and intervertebral level, showing that r was -0.57 and *P* < 0.001, which indicated that distance D decreases as the number of intervertebral increases. Considering the fact that a shorter distance D indicates a higher risk of nerve root injury [25], L4–5 have the higher risk of injury during cage placement. The distance D of L3–4 and L4–5 intervertebral space was greater in men than in women, which suggested that women are at greater risk for nerve root damage in these segments.

## Conclusions

The 3D COSMIC sequences can be used for imaging anatomical assessment before OLIF surgery. The surgical working channel of OLIF surgery is affected by the vertebral body segment, the thickness of the psoas major muscle, sex, and age. In preoperative planning, the 3D COSMIC sequence can be used to measure the relevant parameters mentioned above to optimize the planned surgical approach.

## Data Availability

The datasets generated and analyzed during the present study are available from the corresponding author on reasonable request.

## Abbreviations

GE: General electric company
ICC: Intraclass correlation coefficient
LDD: Lumbar degenerative disease
LIF: Lumbar interbody fusion
OLIF: Oblique lateral lumbar interbody fusion
one-way ANOVA: One-way analysis of variance

## References

[1] Kai W, Cheng C, Yao Q, Zhang C, Jian F, Wu H (2022). Oblique lumbar interbody fusion using a stand-alone construct for the treatment of adjacent-segment lumbar degenerative disease. Front Surg.

[2] Heo DH, Kim JY, Park JY, Kim JS, Kim HS, Roh J, Park CK, Chung H (2022). Clinical experiences of 3-dimensional biportal endoscopic spine surgery for lumbar degenerative disease. Oper Neurosurg.

[3] Zhao L, Xie T, Wang X, Yang Z, Pu X, Zeng J (2022). Whether anterolateral single rod can maintain the surgical outcomes following oblique lumbar interbody fusion for double-segment disc disease. Orthop Surg.

[4] Mayer HM (1997). A new microsurgical technique for minimally invasive anterior lumbar interbody fusion. Spine.

[5] Li JC, Xie TH, Zhang Z, Song ZT, Song YM, Zeng JC (2022). The mismatch between bony endplates and grafted bone increases screw loosening risk for olif patients with alsr fixation biomechanically. Front Bioeng Biotechnol.

[6] Fan W, Yang G, Zhou T, Chen Y, Gao Z, Zhou W, Gu Y (2022). One-stage freehand minimally invasive pedicle screw fixation combined with mini-access surgery through OLIF approach for the treatment of lumbar tuberculosis. J Orthop Surg Res.

[7] Chandra VVR, Bc MP, Hanu TG, Kale PG (2022). Comparison between oblique lumbar interbody fusion (OLIF) and minimally invasive transforaminal lumbar interbody fusion (MISTLIF) for lumbar spondylolisthesis. Neurol India.

[8] Li GQ, Tong T, Wang LF (2022). Comparative analysis of the effects of OLIF and TLIF on adjacent segments after treatment of L4 degenerative lumbar spondylolisthesis. J Orthop Surg Res.

[9] Zhang X, Guo Y, Li Y (2022). Comparison of the clinical efficacy of two fixation methods combined with OLIF in the treatment of lumbar spondylolisthesis in adult patients. J Orthop Surg Res.

[10] Zhou J, Zhou L, Liu C, Yuan C, Wang J (2021). CT value of vertebral body predicting Cage subsidence after stand-alone oblique lumbar interbody fusion. Zhongguo Xiu Fu Chong Jian Wai Ke Za Zhi.

[11] Nagamatsu M, Ruparel S, Tanaka M, Fujiwara Y, Uotani K, Arataki S, Yamauchi T, Takeshita Y, Takamoto R, Tanaka M, Moriue S (2021). Assessment of 3D lumbosacral vascular anatomy for OLIF51 by non-enhanced MRI and CT medical image fusion technique. Diagnostics.

[12] Kugimiya S, Kawasaki H, Kaneko K, Sakai M, Ikeda Y (2012). Fundamental study of three-dimensional coherent oscillatory state acquisition for the manipulation of image contrast: 3D-COSMIC in the spinal region. Nihon Hoshasen Gijutsu Gakkai Zasshi.

[13] An JW, Kim HS, Raorane HD, Hung WP, Jang IT (2022). Postoperative paraspinal muscles assessment after endoscopic stenosis lumbar decompression: magnetic resonance imaging study. Int J Spine Surg.

[14] Amakawa T, Shinohe T, Tominaga S, Honda T, Fukumaru M, Sasaki J (2010). Fundamental study of the fat-suppressed three-dimensional coherent oscillatory state acquisition for the manipulation of image contrast (3D-COSMIC) sequence in the knee joint cartilage. Nihon Hoshasen Gijutsu Gakkai Zasshi.

[15] Ricciardi L, Piazza A, Capobianco M, Della Pepa GM, Miscusi M, Raco A, Scerrati A, Somma T, Lofrese G, Sturiale CL (2021). Lumbar interbody fusion using oblique (OLIF) and lateral (LLIF) approaches for degenerative spine disorders: a meta-analysis of the comparative studies. Eur J Orthop Surg Traumatol.

[16] Davis TT, Hynes RA, Fung DA, Spann SW, MacMillan M, Kwon B, Liu J, Acosta F, Drochner TE (2014). Retroperitoneal oblique corridor to the L2–S1 intervertebral discs in the lateral position: an anatomic study. J Neurosurg Spine.

[17] Chung HW, Lee HD, Jeon CH, Chung NS (2021). Comparison of surgical outcomes between oblique lateral interbody fusion (OLIF) and anterior lumbar interbody fusion (ALIF). Clin Neurol Neurosurg.

[18] Silvestre C, Mac-Thiong JM, Hilmi R, Roussouly P (2012). Complications and morbidities of mini-open anterior retroperitoneal lumbar interbody fusion: oblique lumbar Interbody fusion in 179 patients. Asian Spine J.

[19] Hao J, Yan C, Liu S, Tu P (2018). Effect of bone graft granule volume on postoperative fusion after lumber spinal internal fixation: A retrospective analysis of 82 cases. Pak J Med Sci.

[20] Tsahtsarlis A, Wood M (2012). Minimally invasive transforaminal lumber interbody fusion and degenerative lumbar spine disease. Eur Spine J.

[21] Moser M, Amini DA, Jones C, Zhu J, Okano I, Oezel L, Chiapparelli E, Tan ET, Shue J, Sama AA, Cammisa FP, Girardi FP, Hughes AP (2022). The predictive value of psoas and paraspinal muscle parameters measured on MRI for severe cage subsidence after standalone lateral lumbar interbody fusion. Spine J.

[22] Qian MP, Dong MR, Li J, Kang F (2022). The duration of chronic low back pain is associated with acute postoperative pain intensity in lumbar fusion surgery: a prospective observational study. BMC Anesthesiol.

[23] Zhang X, Wu H, Chen Y, Liu J, Chen J, Zhang T, Zhou Z, Fan S, Dolan P, Adams MA, Zhao F (2021). Importance of the epiphyseal ring in OLIF stand-alone surgery: a biomechanical study on cadaveric spines. Eur Spine J.

[24] Chen X, Chen J, Zhang F (2019). Imaging anatomic research of oblique lumbar interbody fusion in a chinese population based on magnetic resonance. World Neurosurg.

[25] Sugandhavesa N, Kritworakarn N, Rojdumrongrattana B, Sarasombath P, Liawrungrueang W (2022). Spinal nerve compression after malunion of vertical sacrum fractures. Int J Surg Case Rep.

